# Validation of a new predictive risk model: measuring the impact of the major modifiable risks of death for patients and populations

**DOI:** 10.1186/s12963-015-0059-8

**Published:** 2015-10-01

**Authors:** Stephen S. Lim, Emily Carnahan, Eugene C. Nelson, Catherine W. Gillespie, Ali H. Mokdad, Christopher J. L. Murray, Elliott S. Fisher

**Affiliations:** Institute for Health Metrics and Evaluation, University of Washington, 2301 5th Ave., Suite 600, Seattle, WA 98121 USA; The Dartmouth Institute for Health Policy and Clinical Practice, Dartmouth Medical School, Lebanon, NH USA

## Abstract

**Background:**

Modifiable risks account for a large fraction of disease and death, but clinicians and patients lack tools to identify high risk populations or compare the possible benefit of different interventions.

**Methods:**

We used data on the distribution of exposure to 12 major behavioral and biometric risk factors inthe US population, mortality rates by cause, and estimates of the proportional hazards of risk factor exposure from published systematic reviews to develop a risk prediction model that estimates an adult’s 10 year mortality risk compared to a population with optimum risk factors. We compared predicted risk to observed mortality in 8,241 respondents in NHANES 1988-1994 and NHANES 1999-2004 with linked mortality data up to the end of 2006.

**Results:**

Predicted risk showed good discrimination with an area under the receiver operating characteristic (ROC) curve of 0.84 (standard error 0.01) for women and 0.84 (SE 0.01) for men. Across deciles of predicted risk, mortality was accurately predicted in men ((*Χ*^2^ statistic = 12.3 for men, p=0.196) but slightly overpredicted in the highest decile among women (*Χ*^2^ statistic = 22.8, p=0.002). Mortality risk was highly concentrated; for example, among those age 30-44 years, 5.1 % (95 % CI 4.1 % - 6.0 %) of the male and 5.9 % (95 % CI 4.8 % - 6.9 %) of the female population accounted for 25 % of the risk of death.

**Conclusion:**

The risk model accurately predicted mortality in a representative sample of the US population and could be used to help inform patient and provider decision-making, identify high risk groups, and monitor the impact of efforts to improve population health.

**Electronic supplementary material:**

The online version of this article (doi:10.1186/s12963-015-0059-8) contains supplementary material, which is available to authorized users.

## Introduction

The aim of medicine is to reduce the burden of disease [1]. This aim can be achieved by taking actions to promote health and prevent health problems or treat diseases and disabilities after they impose their burden on patients and populations. The evidence suggests a relatively small number of modifiable risks account for a large fraction of the burden of chronic diseases and premature death in the United States as well as the developed world [2–4]. Poor health due to modifiable risks and the costs of treating the resulting disease and injury threaten the affordability of health care. Efforts at prevention or disease modification require not only accurate information on modifiable risks but also the availability of valid, reliable, practical, and actionable measures of these modifiable risks so that those at risk can be identified and interventions appropriately targeted [5–8].

Substantial progress has been made on both fronts in recent years. The Global Burden of Disease initiative has completed systematic reviews identifying and quantifying the modifiable risks of death, disease, and disability in developing and developed countries [9, 10]. Many useful health risk measures have also been developed. Most, however, focus on specific diseases [11, 12] or families of related diseases, such as the widely used Framingham cardiovascular risk index, [13] or on patients in specific care settings who may be at risk for rapid deterioration, such as the APACHE score for intensive care patients [14] or risk indices for frail elderly patients who are hospitalized and may be at risk for decubitus ulcers [15]. Interest in measures of general health risks is also substantial, and many employers and some health systems have adopted health risk appraisals (HRAs) to help their health promotion and disease prevention initiatives. Existing HRAs, however, are based on risk models that have not been validated and published in the literature, or have “black box” scoring algorithms that are not open to scrutiny [16, 17].

To address these limitations, we developed a new, non-proprietary, health risk model based on the most recently available systematic reviews of the modifiable risks of death in order to predict all-cause mortality for adults in the United States. In this report, we describe the validation of this model in a sample of US adults. The findings suggest that the risk prediction model could help individuals and clinicians by allowing them to identify and compare potential clinical and behavioral interventions, while allowing those responsible for defined populations (such as primary care practices, accountable care organizations, health plans, and employers) not only to identify those individuals at greatest risk but also to track changes in health risks over time.

## Methods

The risk model computes an individual’s total risk of mortality over the next 10 years based on exposure to 12 major risk factors (Table 1) for adults aged 30 years or older. These risk factors were included based on reviews of the scientific literature and represent a parsimonious set of the most substantial, modifiable risk factors that contribute to the probability of dying [2]. All risk factors selected had to be (a) actionable: subject to modification by clinical or behavioral interventions, (b) substantial: contribute at least 0.20 years to mortality risk, and (c) evidence-based: supported by recent meta-analyses [9]. We envisioned that the full survey instrument (provided in Additional file 1) could be completed in multiple settings, ranging from clinical visits to online surveys.

**Table 1:** List of risk factors with the corresponding exposure metric

Risk factor	Exposure metric
Excess body weight	Body mass index (kg per m^2^)
High blood pressure	Systolic blood pressure (mmHg)
High cholesterol	LDL cholesterol (mg/dl)
High blood glucose	Fasting plasma glucose (mg/dl)
Seat belts	How often a seat belt is worn:
	• Always or does not drive or ride in a car
	• Nearly always
	• Sometimes
	• Seldom
	• Never
Tobacco use	Three smoking categories:
	• Non-smoker
	• Current smoker
	• Former smoker
Alcohol use	Includes both average consumption and pattern of drinking (binge drinking)
	Average consumption:
	• Abstainer not having had a drink containing alcohol in the last 30 days;
	• 0–19.99 g of pure alcohol daily (females) and 0–39.99 g (males)
	• 20–39.99 g (females) and 40–59.99 g (males);
	• ≥40 g (females) and ≥60 g (males)
	Binge drinking was defined as having at least one occasion of five or more drinks in the last month (men) or four or more drinks in the last month (women)
Physical activity	Based on physical activity during the past 30 days:
	• Inactive, no moderate or vigorous physical activity;
	• Low-active, <2.5 h/wk of moderate activity or <600 MET min/wk;
	• Moderately active: either ≥2.5 h/wk of moderate activity or ≥1 h of vigorous activity; and ≥600 MET min/wk;
	• Highly active: ≥1 h/wk of vigorous activity and ≥1,600 MET min/wk.
Fruit intake	Dietary fruit intake over the past 30 days (average grams per day)
Vegetable intake	Dietary vegetable intake over the past 30 days (average grams per day)
Omega-3 fatty acids intake	Dietary omega-3 fatty acids during the past 30 days (average milligrams of eicosapentaenoic acid (EPA) and docosahexaenoic acid (DHA) per day)
Nut intake	Dietary nut and seed intake, including peanut butter during the past 30 days (average grams per day)

### Risk score development and calculation

**Fig. 1:**
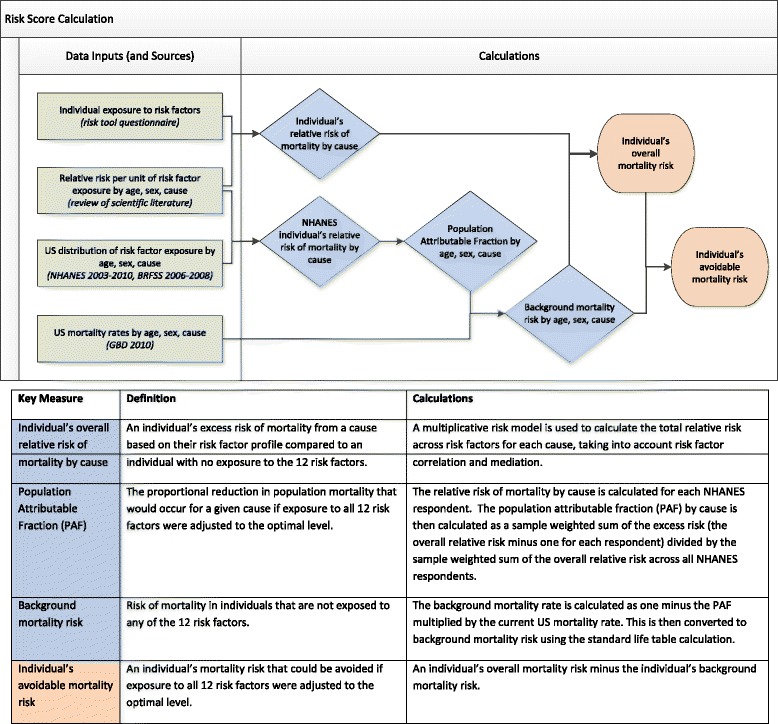
Risk score calculation flowchart: data inputs, sources, and calculations

Figure 1 provides an overview of the data sources and calculations involved in computing a risk score. We briefly summarize the methods below and offer further technical details in Additional file 2.

#### Overall mortality risk

An individual’s risk of mortality is determined by first calculating, for each cause of death separately, the individual’s overall relative risk of mortality compared to no exposure to the 12 risk factors. We used previously published systematic reviews to determine the relative risk per unit increase in exposure to the 12 risk factors by age and sex (Additional file 2). A multiplicative risk model was used to calculate the relative risk of mortality by cause across the 12 risk factors. The model takes into account risk factor correlation (individuals having higher/lower exposure to multiple risk factors due to common socioeconomic or behavioral determinants), and risk mediation (part of the risk associated with factors such as obesity may be mediated through other risk factors such as blood pressure). An individual’s relative risk of mortality by cause is multiplied by the annual background mortality risk by cause to estimate an individual’s overall mortality risk.

#### Avoidable mortality risk

An individual’s avoidable mortality risk, i.e., the mortality risk that could be avoided by reducing exposure to the 12 risk factors to their optimum level, is calculated as the individual’s overall mortality risk less the individual’s background mortality risk based on age and sex. The background mortality risk is an estimate of the risk of mortality over the next 10 years for an individual of the same age and sex who is not exposed to any of the 12 risk factors. We use the currently observed age- and sex-specific background mortality risk to predict an individual’s future background risk of mortality following standard life-table methodology; [18, 19] that is, a woman currently aged 55 is exposed to the background mortality risk of 55-year-old females for the next year, and the background mortality risk of 56-year-old females in the subsequent year, and so on. An individual’s relative risk of mortality by cause is assumed to be constant over all future periods. An individual’s overall risk of mortality from all causes over the next 10 years and their remaining life expectancy are calculated using the standard competing risk model [20].

#### Background mortality risk by cause

To determine the background mortality risk by cause for the current period we combined information on (a) the current distribution of exposure to the 12 risks by age and sex using data from the National Health and Nutrition Examination Survey (NHANES, 2003–2010) and the Behavioral Risk Factor Surveillance System (BRFSS, 2006–2008); (b) age-, sex-, and cause-specific mortality rates in 2010 from the Global Burden of Disease Study 2010; [21] and (c) relative risks by age, sex, and cause associated with exposure to the 12 risk factors from systematic reviews. The mortality rates have been adjusted for errors in cause of death assignment using previously described methods [22].

Currently observed age-, sex-, and cause-specific mortality rates represent the rate at which individuals of that age and sex group will die from a cause in a given year. These rates reflect the current exposure to risk factors in the population and their hazardous effects on mortality. The fraction of mortality that is due to current exposure to the 12 risk factors can therefore be determined by calculating a population attributable fraction (PAF) by age, sex, and cause; this was done by calculating the overall relative risk due to the 12 risk factors for each respondent in NHANES (2003–2010), taking into account risk factor correlation and mediation [3]. The PAF for each age, sex, and cause is calculated as the sample weighted sum of the excess risk, i.e., the relative risk minus one, divided by the sample weighted sum of the relative risk across all NHANES respondents. To address selection bias we imputed missing risk factor values using multiple imputations and took the average across the 10 imputations. The background mortality rate by cause is calculated as one minus the PAF multiplied by the current mortality rate. Mortality rates were converted to annual probabilities of dying using the standard life table calculation [18, 19].

### Risk score validation

We performed an out-of-sample validation test using established methods and NHANES linked mortality data through December 31, 2006, for respondents interviewed between 1988 and 1994 and between 1999 and 2004. These data were not used in the construction of the risk score. For each individual in the cohort we calculated the predicted risk of mortality over the available follow-up time period up to 10 years. The validation assessed (a) *discrimination*: the ability of the risk model to distinguish between those who die during the follow-up period and those who survive by calculating the area under the curve (AUC) for the receiver operating characteristic curve; [23] and (b) *calibration*: the ability of the risk model to predict the observed level of risk across deciles of the population using the Hosmer-Lemeshow *Χ*^2^ statistic. The validation was performed for men and women and by age group.

### Impact on life expectancy and distribution of risk

We examined life expectancy and distribution of risk for the NHANES 2003–2010 cohort, a sample that is representative of the US population. We calculated the increase in life expectancy that would result from reducing exposure to the optimum distribution for each individual risk factor as well as the 12 risk factors jointly as previously described [3]. We also used the risk model to estimate the 10-year total and avoidable risk of death for each respondent from NHANES 2003–2010 and present results on the concentration of risk.

All analyses were conducted in Stata 11 (Stata Corporation, Texas).

## Results

### Accuracy of the mortality prediction: Risk model validation

**Fig. 2:**
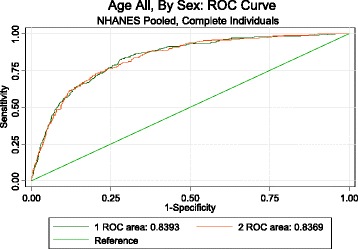
Receiver operator characteristic (ROC) curve for risk score (NHANES 1988–1994 and 1999–2004). Note: Males: green curve, Females: red curve

**Fig. 3:**
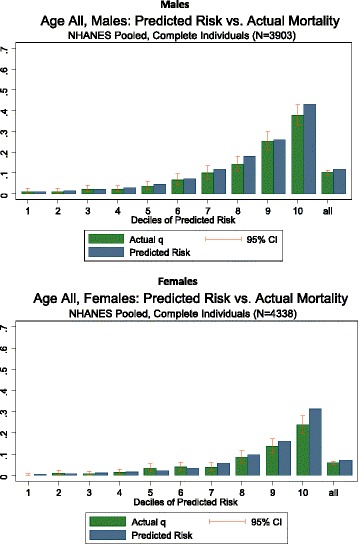
Comparison of predicted risk of death against observed risk of death (NHANES 1988–1994 and 1999–2004)

Additional file 1 summarizes the characteristics of the 8,241 NHANES respondents included in the validation dataset. By the end of 2006, 696 deaths (419 men and 277 women) occurred in this cohort. The risk model was able to discriminate well between individuals who died and those who survived, with an area under the curve (AUC) of 0.84 (SE = 0.01) for women and 0.84 (SE = 0.01) for men (Fig. 2) for deaths from any cause. The risk model also accurately predicted the risk of death across deciles (Fig. 3) among men (*Χ*^2^ = 12.3, *p* = .196). Risk was slightly overestimated in the highest risk decile among women (*Χ*^2^ = 22.8, *p* = .002). These results indicate that the risk model is sufficiently accurate for use as a predictor of mortality risk.

### What matters most: Impact of specific risk factors on US and individual mortality risk

Table 2 shows the estimated effect on life expectancy of shifting risk exposures to their optimum distribution (i.e., no excess risk) within the NHANES 2003–2010 cohort, a proxy for the US population. The combined impact of the 12 risk factors is substantial; life expectancy would increase by more than nine years among men and more than eight years among women. For the US adult population as a whole, the model indicates that tobacco smoking, high blood pressure, and excess body weight are the modifiable risk factors with the largest effect on current adult life expectancy. The model also enables different risks to be identified, quantified, and compared for counseling individual patients.

**Table 2:** Life expectancy gains in the US population (in years) by removing risk factors

	Smoking	High blood pressure	Excess body weight	High blood sugar	High cholesterol	Low physical activity	Low nut intake	Low vegetable intake	Low fruit intake	Low omega-3 intake	Alcohol intake	Inadequate seat belt use	Joint effects
Male	3.20	2.50	2.30	1.57	1.33	1.27	0.87	0.76	0.72	0.62	0.58	0.24	9.59
Female	2.39	2.92	2.21	1.38	0.92	1.39	0.72	0.70	0.61	0.54	0.40	0.23	8.98

### Identifying high-risk patients: Distribution of risk in a population

**Fig. 4:**
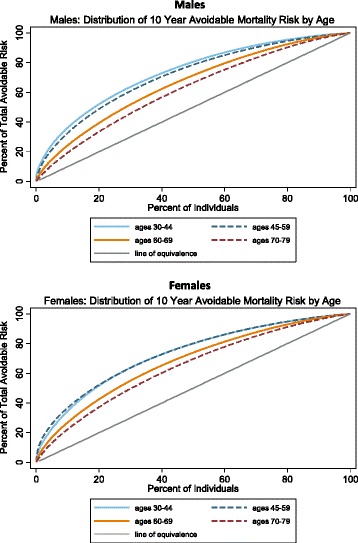
Distribution of avoidable risk of mortality in the United States by age and sex (NHANES 2003–2010)

The avoidable risk of death is heavily concentrated in a relatively small fraction of the population, particularly at younger ages (Fig. 4). Among males aged 30 to 44 years, 5.1 % (95 % CI 4.1–6.0 %) of males account for 25 % of the avoidable risk of death in that age group. This is similar in females aged 30 to 44 years, with 5.9 % (95 % CI 4.8–6.9 %) of females accounting for 25 % of the avoidable risk of death. In general, as the population ages, this fraction tends to increase. For example, among males aged 70 to 79, 13.9 % (95 % CI 11.1–16.6 %) of males account for 25 % of avoidable mortality risk in that age group, and among females aged 70 to 79 years, 11.7 % (95 % CI 9.2–14.1 %) of females account for 25 % of avoidable mortality risk in that age group.

## Discussion

While the need for accurate and actionable information on modifiable health risks is well recognized, the risk models currently available have important limitations, largely because they either focus on a specific, important cause of death [24] or because they are based on proprietary algorithms that may not be based on the most up-to-date evidence and have not been validated in the general US population. Individuals, clinicians, and others therefore currently lack a broadly available, evidence-based tool that would support more accurate identification of risks, assessment and comparison of the potential impact for individuals of different risk-modification strategies, or identification of high-risk subgroups of the population. The risk model described and validated here attempts to overcome these limitations.

We developed a new risk model based on 12 major behavioral and biometric risks to health that predict an individual’s probability of dying over the next 10 years compared to a population with an optimum distribution of risk factors. The model is based on current scientific evidence on risks to health and contemporaneous, high-quality data on the distribution of risk exposures and mortality rates in the US population. The model has excellent discrimination in a sample of the US population [23]. The excellent and widely used Framingham Index [24–26] typically has areas under the curve of between 0.75 and 0.80 for predicting cardiovascular endpoints only [27]. This newly developed model has areas under the curve of greater than 0.80 for both men and women for the more difficult task of predicting mortality from any cause and is constructed using the best available evidence on the major modifiable causes of mortality.

The risk model offers promise as a tool to support individual decision-making. A recent systematic review demonstrates the benefits of providing cardiovascular risk information to individuals for discussion with their families and physicians [28]. The results suggest that if individuals understand the magnitude of their risk, they are more likely to adopt or maintain healthy behaviors. The review also found that information on overall risk is likely to have a greater impact when it is paired directly with education or counseling. As illustrated in Additional file 3, this risk model can facilitate counseling and decision-making by providing a systematic way for patients and clinicians to compare how different behavioral or clinical interventions would likely influence risk. For example, the importance of smoking cessation for most smokers becomes obvious. The risk model can show patients how they might avoid medication use by increasing physical activity or reducing weight, or conversely, the lost benefits of failing to take prescribed medications. The risk model also takes into account the effect of behavioral modification on other biological risk factors included in the risk score, e.g., the effect of reductions in body weight on blood pressure. Presenting accurate, holistic, and balanced information about the risks that patients face, in conjunction with counseling about the importance of different options for change, could help align decision-making with patients’ preferences – an important national aim [28]. Implementing the model in a way that provides an attractive and accessible tool for the general population, e.g., through Internet or phone-based applications, would also provide a way for individuals to self-assess their risk and encourage contacts with healthcare providers to decrease risk.

Broad adoption of the risk model could offer other important benefits. First, clinical practices, health systems, and workplace health programs that obtain completed surveys from their populations can accurately stratify people according to their level of risk and develop epidemiologically informed programs to reduce risks. In the highest-risk subpopulation, individualized multiple-risk-factor interventions, such as case management and health coaching, could be a wise investment [29]. Second, the measure could contribute to clinical and public health research by providing a validated composite endpoint for clinical trials of multiple-risk-factor intervention programs. Third, the measure offers a potential improvement over current quality indicators that focus largely on intermediate outcomes (e.g., levels of blood sugar, blood pressure, and cholesterol) that have little intuitive meaning to patients and encourage well-recognized hazards among providers. For example, if the proportion of diabetics with well-controlled HbA1c is used as a quality measure, some may be encouraged to label those with pre-diabetes as diabetic (inflating the denominator), and treatment efforts may be focused on those with mild disease, in whom achieving target levels is easier but less important. Performance measured on the basis of improvement in predicted risk could give greater credit to meaningful progress for those at greatest risk, even if specified targets were not achieved. In addition, the risk measure would in all likelihood be more parsimonious (i.e., one broad measure of risk status versus many narrow measures) [30]. Finally, if broadly adopted within specific geographic regions and mapped in ways that preserve confidentiality, the measure could provide a basis for collaboration among providers and community stakeholders on initiatives to improve population health.

At the same time, the risk model has important limitations. We made judgments about which risks to include. For example, we did not include depression, believing that screening and intervention (especially for those at risk of suicide) has a different and more pressing time horizon, and we judged trans fats to be akin to an environmental risk that is difficult for an individual to control or modify. The risk model is based on the average American, and, although differential risk exposure explains a large fraction of geographic, racial, and socioeconomic factors, there are likely to be residual differences in the underlying risk of death between these groups. Although the proportional hazards of risk exposure are largely generalizable across different populations, [31] several risk factors are based on self-report, and these responses may not be accurate or comparable across populations. While all included risks have strong evidence as predictors of mortality, the strength of evidence for risk factor modification on reduction in mortality differs across risks, with a greater body of evidence available for risk factors such as high blood pressure. Related to this, some of the evidence for these risk factors is based on observational studies, which are prone to potential confounding. Although we split the development and validation datasets, our use of the same broad data source (NHANES) for the analysis may have caused us to overestimate the predictive validity. The risk score appears to be mainly driven by cardio-metabolic factors and has not yet been validated either for specific clinical populations (e.g., cancer, arthritis, dementia) or for different racial, ethnic, or socioeconomic groups. The risk score also does not include a prediction of morbidity, which is an important consideration particularly among older-aged individuals, nor does it presently include a quantification of the potential adverse effects, for example of medication, to reduce exposure to risk factors. It will be important for patients and clinicians to understand these limitations, both about the relative magnitude of specific risks and the potential benefits of risk factor modification and to conduct further validation studies and to update the model as new evidence accumulates. Finally, while the focus of the use of the risk score presented in this paper is on individual-level modification, this should also be balanced against population-wide approaches for reducing risk exposure.

Some of these limitations can be addressed or mitigated by implementing the risk model in diverse practices and populations and by linking respondents’ original and subsequent risk factor scores to data on survival. This would facilitate continued improvement and validation of the model. This will be increasingly possible as electronic health record-enabled environments are adopted for large patient populations [32].

## Conclusion

The need to balance *downstream* treatment of diseases with *upstream* prevention is well recognized [6, 17, 29]. Legislative and other upstream actions are now a cornerstone of risk reduction and in some countries they play a key role in reducing non-communicable diseases. Patients, clinicians, and employers are faced with a wide array of commercial health risk appraisal tools that aim to catalyze prevention and health promotion. Incorporating a standardized, validated, freely available, transparent, and continuously refined method to measure, summarize, and track the most important modifiable risks of death within these tools would offer benefits to patients, clinicians, and policymakers.

## Additional files

Additional file 1:
**Web Table 1.** Characteristics of the validation cohort (NHANES 1988–1994 and 1999–2004) (DOCX 21 kb) Additional file 2:
**Web Appendix B: Technical information on risk score development and derivation.** (DOCX 49 kb) Additional file 3:
**Web Appendix C: Example of risk calculator applied to a particular person: male age 35, smoker, uncontrolled blood pressure, limited physical activity.** (DOCX 246 kb)
