# Supplementary material for: Validation of a new predictive risk model: measuring the impact of the major modifiable risks of death for patients and populations
Source: Popul Health Metr. 2015 Oct 1;13:27. doi: 10.1186/s12963-015-0059-8 (PMC4591717; doi:10.1186/s12963-015-0059-8)
Supplement: Additional file 1: — Web Table 1. Characteristics of the validation cohort (NHANES 1988–1994 and 1999–2004) (DOCX 21 kb) [file 12963_2015_59_MOESM1_ESM.docx]

**Web Table 1. Characteristics of the validation cohort (NHANES 1988-1994 and 1999-2004)**

|  |  | **NHANES 1988-1994, 1999-2004 cohort** | | | |
| --- | --- | --- | --- | --- | --- |
|  |  | **Males (N=3903)** | | **Females (N=4338)** | |
| **Demographic characteristics** | | **N (%)** | | **N (%)** | |
| ***Age Categories*** | |  |  |  |  |
|  | 30-44 years | 1,426 | (37) | 1,780 | (41) |
|  | 45-59 years | 1,039 | (27) | 1,180 | (27) |
|  | 60-69 years | 856 | (22) | 869 | (20) |
|  | 70+ years | 582 | (15) | 509 | (12) |
| ***Race/Ethnicity*** | |  |  |  |  |
|  | Non-Hispanic White | 1,911 | (49) | 2,035 | (47) |
|  | Non-Hispanic Black | 841 | (22) | 1,040 | (24) |
|  | Mexican American | 968 | (25) | 1,033 | (24) |
|  | Other Race (incl. Multi-Racial) | 70 | (2) | 80 | (2) |
|  | Other Hispanic | 113 | (3) | 150 | (3) |

**Web Table 1 (cont). Characteristics of the validation cohort (NHANES 1988-1994 and 1999-2004)**

|  |  | **NHANES 1988-1994, 1999-2004 cohort** | | | | | | | |
| --- | --- | --- | --- | --- | --- | --- | --- | --- | --- |
|  |  | **Males** | | | | **Females** | | | |
| ***Risk Factors*** | | **N (%)** | | **Mean** | **Std Dev** | **N (%)** | | **Mean** | **Std Dev** |
| ***LDL cholesterol (mmol/L)*** | | 3,903 | (100) | 3.4 | 0.9 | 4,338 | (100) | 3.3 | 1.0 |
| ***Fasting plasma glucose (mmol/L)*** | | 3,903 | (100) | 5.9 | 1.7 | 4,338 | (100) | 5.7 | 1.8 |
| ***Body mass index (kg/m^2)*** | | 3,903 | (100) | 27.7 | 5.0 | 4,338 | (100) | 28.7 | 6.6 |
| ***Systolic blood pressure (mmHg)*** | | 3,903 | (100) | 127.3 | 17.6 | 4,338 | (100) | 124.2 | 20.1 |
| ***Fruit intake (grams/day)*** | | 3,903 | (100) | 119.5 | 204.6 | 4,338 | (100) | 127.0 | 185.9 |
| ***Vegetable intake (grams/day)*** | | 3,903 | (100) | 133.1 | 181.0 | 4,338 | (100) | 132.8 | 161.4 |
| ***Omega 3 fatty acids (mg/day)*** | | 3,903 | (100) | 102.0 | 314.1 | 4,338 | (100) | 83.7 | 283.9 |
| ***Nut intake (grams/day)*** | | 3,903 | (100) | 4.1 | 14.0 | 4,338 | (100) | 3.5 | 13.5 |
| ***Smoking*** |  |  |  |  |  |  |  |  |  |
|  | Non-Smoker | 1,339 | (34) |  |  | 2,496 | (58) |  |  |
|  | Former Smoker | 1,442 | (37) |  |  | 961 | (22) |  |  |
|  | Current Smoker | 1,122 | (29) |  |  | 881 | (20) |  |  |
| ***Binge drinking*** | |  |  |  |  |  |  |  |  |
|  | Yes | 866 | (22) |  |  | 198 | (5) |  |  |
|  | No | 3,037 | (78) |  |  | 4,140 | (95) |  |  |
| ***Alcohol use*** | |  |  |  |  |  |  |  |  |
|  | Abstainer | 1,336 | (34) |  |  | 2,376 | (55) |  |  |
|  | Group I | 2,295 | (59) |  |  | 1,826 | (42) |  |  |
|  | Group II | 166 | (4) |  |  | 103 | (2) |  |  |
|  | Group III | 106 | (3) |  |  | 33 | (1) |  |  |
| ***Physical Activity**** | |  |  |  |  |  |  |  |  |
|  | Inactive | 795 | (42) |  |  | 916 | (46) |  |  |
|  | Low-active | 400 | (21) |  |  | 488 | (24) |  |  |
|  | Moderately active | 452 | (24) |  |  | 390 | (20) |  |  |
|  | Highly active | 266 | (14) |  |  | 206 | (10) |  |  |
| *Physical Activity levels are only available for the 1999-2004 NHANES cohort. | | | | | | | | | |
