# Supplementary material for: Validation of a new predictive risk model: measuring the impact of the major modifiable risks of death for patients and populations
Source: Popul Health Metr. 2015 Oct 1;13:27. doi: 10.1186/s12963-015-0059-8 (PMC4591717; doi:10.1186/s12963-015-0059-8)
Supplement: Additional file 2: — Web Appendix B: Technical information on risk score development and derivation. (DOCX 49 kb) [file 12963_2015_59_MOESM2_ESM.docx]

**Web Appendix B: Technical Information on Risk Score Development and Derivation**

The risk calculator was developed by combining data on the distribution of risk exposure in the US population using data from the National Health and Nutrition Examination Survey (NHANES, 2003-2010) and the Behavioral Risk Factor Surveillance System (BRFSS, 2006-2008) with the underlying age, sex and cause-specific mortality rates from the Global Burden of Disease Study 2010 and a systematic review of the proportional hazards by cause associated with exposure to the 12 risk factors.

We first take into account mediation of distal risk factors by more proximal risk factors that are included in the risk score by adjusting the estimate of the proportional hazard of distal risk factors as follows (Equation 1):

$$\beta_{rc}=\ln((RR_{rc}-1)\cdot(1-\alpha_{rc})+1)$$

where:

*r* denotes each of the 12 risk factors described in Table 1

*c* denotes the cause of mortality

*β* is the log of the relative risk (RR) per unit exposure or category of exposure that represents the total effect of the risk factor, i.e. including both direct and mediated effects

*α* is the proportion of the excess risk that is mediated through more proximal risk factors included in the risk score

For each risk factor and cause we obtained relative risks (RRs) by age and sex and the threshold exposure level from systematic reviews and meta-analyses of most recent epidemiologic studies (Annex 1). For distal risk factors we reviewed the scientific literature to determine the proportion of excess risk that is mediated from more proximal risk factors; we include mediated effects from body mass index (BMI), physical activity, and omega-3 fatty acids.

After adjusting the relative risks for mediated effects, the total relative risk of mortality for each cause for the individual is computed using a multiplicative model as follows (Equation 2):

where:

*i* denotes the individual

*c* denotes the cause of mortality

*R* denotes the total number of risk factors included in the equation, i.e. 12

*r* denotes each of the 12 risk factors described in Table 1

*β* is the log of the relative risk (RR) per unit exposure or category of exposure

*L* is the current exposure level for the individual

*T* is the threshold exposure level

For each annual time period over the next 10 years, i.e. from the current year (t=0) to the tenth time period (t=9), we calculated the individual’s probability of dying during that time period by cause (Equation 3):

$$TQ_{iasct}=RR_{ic}\cdot CQ_{a+t,sc}$$

where:

*TQ_iasct_* is the probability of dying from cause *c* during the one-year time period *t* for individual *i*, who is currently aged *a* years and of sex *s*

*CQ_a+t,sc_* is the probability of dying due to cause *c* that is not attributable to any of the 12 risk factors for the group currently aged *a+t* and of sex *s*

*RR_ic_* is the total relative risk of mortality for cause *c* that represents the combined effects of exposure to the 12 risk factors compared to no exposure for individual *i*

The total probability of an individual dying from all *C* causes during each one year time period is then computed using the standard competing risk model (Equation 4):

$$TQ_{iast}=1-\prod_{c}^{C} (1-TQ_{iasct})$$

where:

*TQ_iast_* is the probability of dying from all causes during the one-year time period *t* for individual *i*, who is currently aged *a* years and of sex *s*

*TQ_iasct_* is the probability of dying from cause *c* during the one-year time period *t* for individual *i*, who is currently aged *a* years and of sex *s*

The 10-year probability of dying from all causes is then calculated using standard life-table calculations as (Equation 5):

$$TQ10_{ias}=TQ_{ias,t=0}+TQ_{ias,t=1}\cdot\left( 1-TQ_{ias,t=0}\right)+TQ_{ias,t=2}\cdot\left( 1-TQ_{ias,t=0}\right)\cdot\left( 1-TQ_{ias,t=1}\right)+....$$

where:

*TQ10_ias_* is the probability of dying from all causes over the next 10 years for individual *i*, who is currently aged *a* years and of sex *s*

*TQ_iast_* is the probability of dying from all causes during the one-year time period *t* for individual *i*, who is currently aged *a* years and of sex *s*

To determine an individual’s probability of dying that is avoidable by modifying their exposure to the 12 risk factors, we first compute the background or counterfactual probability of dying (Equation 6 and 7):

$$CQ_{iast}=1-\prod_{c}^{C} (1-CQ_{iasct})$$

$$CQ10_{ias}=CQ_{ias,t=0}+CQ_{ias,t=1}\cdot\left( 1-CQ_{ias,t=0}\right)+CQ_{ias,t=2}\cdot\left( 1-CQ_{ias,t=0}\right)\cdot\left( 1-CQ_{ias,t=1}\right)+....$$

where:

*CQ_iast_* is the probability of dying from all causes that is not attributable to the 12 risk factors during the one-year time period *t* for individual *i*, who is currently aged *a* years and of sex *s*

*CQ_iasct_* is the probability of dying from cause *c* that is not attributable to the 12 risk factors during the one-year time period *t* for individual *i*, who is currently aged *a* years and of sex *s*

*CQ10_ias_* is the probability of dying from all causes over the next 10 years that is not attributable to the 12 risk factors for individual *i*, who is currently aged *a* years and of sex *s*

The difference between TQ10_ias_ and CQ10_ias_ is the avoidable risk of mortality for the individual.

***Probability of dying by cause that is not attributable to the 12 risk factors***

To determine an individual’s probability of dying related to the 12 risk factors we need to know the underlying population-level probability of dying for each cause that is not attributable to any of the 12 risk factors, i.e. the counterfactual mortality. Population-level age-, sex- and cause-specific mortality rates represent the rate at which individuals of that age and sex group will die from a cause in a given year. These rates reflect the current exposure to risk factors in the population. We can therefore determine the underlying mortality rate that is unrelated to the 12 risk factors by combining disease-specific mortality rates with data on exposure distributions to risk factors and the proportional hazards by cause for each risk factor.

We first computed the total relative risk due to the 12 risk factors for each observation (Equation 2) in a representative sample of the US population by sex, age group and cause taking into account risk mediation (Equation 1). We take into account correlation in risk factor exposure by using data from the National Health and Nutrition Examination Survey (NHANES, 2003-2010) that measures exposures in the same individuals for all risk factors except for seat belt use. Prior to using the data we first impute missing values for all risk factors and respondents using multiple imputation; we created 10 imputed datasets. For seat-belt use we calculated the national age-and-sex specific mean exposure from the Behavioral Risk Factor Surveillance System (BRFSS) and applied this to each observation from NHANES. This assumes that exposure to seat-belt use is uncorrelated with exposure to other risks. The population attributable fraction (PAF) for each age, sex and cause is then computed by calculating the sample weighted sum of the excess risk divided by the sample weighted sum of the relative risk (Equation 9):

$$PAF_{asc}=\frac{\sum_{i} (RR_{ic}-1)}{\sum_{i} RR_{ic}}$$

where:

*PAF_asc_* is the population attributable fraction for the 12 risk factors for mortality due to cause c for the group aged *a* and of sex *s*

*RR_sc_* is the total relative risk of mortality from cause *c* that reflects exposure to all 12 risk factors for individual *i* aged *a* and of sex *s*

The annual mortality rate that is not due to the 12 risk factors is then given by (Equation 10):

$$CM_{asc}=M_{asc}\cdot\left( 1-PAF_{asc}\right)$$

where:

*CM_asc_* is the mortality rate due to cause c that is not attributable to any of the 12 risk factors for the group aged *a* and of sex *s*

*M_asc_* is the mortality rate due to cause *c* for the group aged *a,* and of sex *s*.

We used mortality rates for 2010 by cause, age and sex from the Global Burden of Disease Study 2010. These have been adjusted for errors in cause of death assignment using previously described methods.^1^ We obtained population estimates for 2010 by age and sex, also from the Global Burden of Disease Study 2010. We used demographic techniques to estimate mortality by cause for 5-year age groups up to age 100 using data from the open ended age group (ages 80+).

We converted mortality rates to an annual probability of dying using the standard life table calculation (Equation 11):

$$CQ_{asc}=\frac{n\cdot CM_{asc}}{(1+(n-{}_{n}a_{x})\cdot CM_{asc})}$$

***Individual mortality risk due to diabetes and alcohol abuse***

Mortality due to diabetes and alcohol abuse is fully attributable to blood glucose and alcohol use, respectively. As a result, the methods described for calculating an individual’s mortality risk for these causes is not applicable, i.e. the counterfactual mortality is zero (this also means that for these two risk factor-disease pairs, total cause-specific mortality (TQ) and avoidable cause-specific mortality (AQ) are equivalent). For these two risk factor-disease pairs, we computed the probability of dying from these causes as follows (Equation 12):

where:

*q_iasc_* is the probability of dying from alcohol or diabetes for individual *i* aged *a,* and of sex *s*.

$\bar{q}_{as}$is the mean probability of dying from alcohol or diabetes for the group aged *a,* and of sex *s*.

is the exponential of the product of the log of the relative risk and the exposure to fasting plasma glucose or alcohol use for individual *i* aged *a,* and of sex *s*.

$e^{\beta x_{ias}}$is exponential of the product of the log of the relative risk and the mean exposure to fasting plasma glucose or alcohol use for the group aged *a,* and of sex *s*.

As relative risks for the effects of fasting plasma glucose and alcohol consumption on diabetes and alcohol abuse respectively are not available we use the relative risk of ischemic heart disease for fasting plasma glucose and the relative risk of liver cirrhosis for alcohol consumption.

Bibliography

1. Naghavi M, Makela S, Foreman K, O’Brien J, Pourmalek F, Lozano R. Algorithms for enhancing public health utility of national causes-of-death data. Popul Health Metrics 2010;8(1):9.

**Annex 1. Summary of studies used to derive relative risks.**

| **Risk Factor** | **Disease Outcome** | **Source of RR** |
| --- | --- | --- |
| Low intake of fruits | IHD | Unpublished meta-analysis of 9 cohorts for GBD 2010 based on He et al. [1] |
|  | Ischemic stroke | Unpublished meta-analysis of 9 cohorts for GBD 2010 based on He et al. [1] |
|  | Hemorrhagic stroke | Unpublished meta-analysis of 5 cohorts for GBD 2010 based on He et al. [1] |
|  | Lung cancer | Meta-analysis of 4 cohort studies [2] |
|  | Esophagus cancer | Analysis of 345,904 subjects in the prospective European Investigation into Cancer and Nutrition (EPIC) [3] |
|  | Mouth and pharynx cancers | Analysis of 345,904 subjects in the prospective European Investigation into Cancer and Nutrition (EPIC) [3] |
|  | Larynx cancer | Analysis of 345,904 subjects in the prospective European Investigation into Cancer and Nutrition (EPIC) [3] |
| Low intake of vegetables | IHD | Unpublished meta-analysis of 9 cohorts for GBD 2010 based on He et al. [1] |
|  | Ischemic stroke | Unpublished meta-analysis of 8 cohorts for GBD 2010 based on He et al. [1] |
|  | Hemorrhagic stroke | Unpublished meta-analysis of 5 cohorts for GBD 2010 based on He et al. [1] |
|  | Mouth and pharynx cancers | Analysis of 345,904 subjects in the prospective European Investigation into Cancer and Nutrition (EPIC) [3] |
|  | Larynx cancer | Analysis of 345,904 subjects in the prospective European Investigation into Cancer and Nutrition (EPIC) [3] |
| Low dietary omega-3 fatty acids | IHD | Unpublished meta-analysis of 22 studies, including RCTs and observational studies, for GBD 2010 [4] |
| Alcohol use | IHD | Meta-analysis of observational studies for non-binge [5,6] and binge drinkers [7] |
|  | Ischemic stroke | Meta-analysis of 35 observational studies [5,8] |
|  | Hemorrhagic stroke | Meta-analysis of 35 observational studies [5,8] |
|  | Hypertensive disease | Overview of observational studies [5,9,10] |
|  | Cardiac arrhythmias | Overview of observational studies [9] |
|  | Breast cancer | Systematic review of epidemiological studies [5,9,10] |
|  | Colorectal cancer | Pooled analysis of 8 prospective cohort studies [11] |
|  | Esophagus cancer | Overview of observational studies [5,9,10] |
|  | Mouth and pharynx cancer | Overview of observational studies [5,9,10] |
|  | Laryngeal cancer | Overview of observational studies [9] |
|  | Liver cancer | Overview of observational studies [5,9,10] |
|  | Selected other cancers | Overview of observational studies [5,10] |
|  | Liver cirrhosis | Overview of observational studies [5,9,10] |
|  | Acute and chronic pancreatitis | Meta-analysis of observational studies [12] |
|  | Road traffic injury deaths | Grand Rapids Study [5,13] |
|  | Falls, homicide and suicide, and other injury deaths | Grand Rapids Study [5,13] |
| Physical inactivity | IHD | Meta-analysis of 20 prospective cohort studies [14] |
|  | Ischemic stroke | Meta-analysis of 8 prospective cohort studies [14] |
|  | Breast cancer | Meta-analysis of 12 prospective cohort and 31 case-control studies [14] |
|  | Colon cancer | Meta-analysis of 11 prospective cohort and 19 case-control studies [14] |
| Tobacco smoking | IHD | ACS CPS-II [15] |
|  | Stroke | ACS CPS-II [15] |
|  | Selected other cardiovascular diseases | ACS CPS-II [15] |
|  | Lung cancer | ACS CPS-II [16] |
|  | Mouth, pharynx, and esophagus cancer | ACS CPS-II [16] |
|  | Stomach cancer | ACS CPS-II [16] |
|  | Liver cancer | ACS CPS-II [16] |
|  | Pancreas cancer | ACS CPS-II [16] |
|  | Cervix uteri cancer | ACS CPS-II [16] |
|  | Bladder cancer | ACS CPS-II [16] |
|  | Leukemia | ACS CPS-II [16] |
|  | Kidney and other urinary cancer | ACS CPS-II [16] |
|  | Chronic obstructive pulmonary disease | ACS CPS-II [17] |
|  | Other respiratory diseases | ACS CPS-II [17] |
|  | Tuberculosis | Meta-analysis of cohort, case-control, and cross-sectional studies [18] |
| High blood glucose | IHD | Unpublished meta-analysis of 3 cohort studies (APCSC, DECODE, ERFC) for GBD 2010 [4] |
|  | Stroke | Unpublished meta-analysis of 3 cohort studies (APCSC, DECODE, ERFC) for GBD 2010 [4] |
|  | Chronic kidney disease | Analysis of 10 studies with 227,746 participants in the Asia-Pacific region [19] |
| High LDL cholesterol | IHD | Unpublished meta-analysis of 2 cohort studies (APCSC, PSC) for GBD 2010 [4] |
|  | Ischemic stroke | Unpublished meta-analysis of 2 cohort studies (APCSC, PSC) for GBD 2010 [4] |
| High blood pressure | IHD | Unpublished meta-analysis of 2 cohort studies (APCSC, PSC) for GBD 2010 [4] |
|  | Ischemic stroke | Unpublished meta-analysis of 2 cohort studies (APCSC, PSC) for GBD 2010 [4] |
|  | Hemorrhagic stroke | Unpublished meta-analysis of 2 cohort studies (APCSC, PSC) for GBD 2010 [4] |
|  | Hypertensive disease | Unpublished meta-analysis of 2 cohort studies (APCSC, PSC) for GBD 2010 [4] |
|  | Atrial fibrillation and flutter | Unpublished meta-analysis of 2 cohort studies (APCSC, PSC) for GBD 2010 [4] |
|  | Rheumatic heart disease | Unpublished analysis of PSC for GBD 2010 [4] |
|  | Cardiomyopathy and myocarditis | Unpublished analysis of PSC for GBD 2010 [4] |
|  | Peripheral vascular disease | Unpublished meta-analysis of 2 cohort studies (APCSC, PSC) for GBD 2010 [4] |
|  | Endocarditis | Unpublished analysis of PSC for GBD 2010 [4] |
|  | Aortic aneurysm | Unpublished analysis of PSC for GBD 2010 [4] |
|  | Other cardiovascular diseases | Unpublished meta-analysis of 2 cohort studies (APCSC, PSC) for GBD 2010 [4] |
|  | Chronic kidney disease | Unpublished meta-analysis of 106 cohorts and 2.7 million individuals [20] |
| High body mass index (BMI) | IHD | Unpublished meta-analysis of 3 cohort studies (APCSC, ERFC, PSC) for GBD 2010 [4] |
|  | Ischemic stroke | Unpublished meta-analysis of 3 cohort studies (APCSC, ERFC, PSC) for GBD 2010 [4] |
|  | Hypertensive disease | Unpublished analysis of PSC for GBD 2010 [4] |
|  | Atrial fibrillation and flutter | Unpublished analysis of PSC for GBD 2010 [4] |
|  | Cardiomyopathy and myocarditis | Unpublished analysis of PSC for GBD 2010 [4] |
|  | Endocarditis | Unpublished analysis of PSC for GBD 2010 [4] |
|  | Peripheral vascular disease | Unpublished analysis of PSC for GBD 2010 [4] |
|  | Other cardiovascular diseases | Unpublished analysis of PSC for GBD 2010 [4] |
|  | Breast cancer | Meta-analysis of 31 prospective cohort studies [21] |
|  | Colon cancer | Meta-analysis of 22 prospective cohort studies in males and 19 in females [21] |
|  | Corpus uteri cancer | Meta-analysis of 19 prospective cohort studies [21] |
|  | Kidney cancer | Meta-analysis of 11 prospective cohort studies in males and 12 in females [21] |
|  | Pancreatic cancer | Meta-analysis of 12 prospective cohort studies in males and 11 in females [21] |
|  | Esophagus cancer | Meta-analysis of 5 studies in males and 3 studies in females [21] |
|  | Gallbladder cancer | Meta-analysis of 4 studies in males and 2 studies in females [21] |
|  | Chronic kidney disease | Unpublished analysis of PSC for GBD 2010 [4] |
| Seat belt use | Road traffic injury deaths | Matched-pair cohort study using a sample of 88,778 cars from the Fatality Analysis Reporting System (FARS) [22] |
| Nut intake | IHD | Meta-analysis of 4 prospective cohort studies [23] |

Cohort abbreviations: Asia-Pacific Cohort Studies Collaboration (APCSC); Diabetes Epidemiology: Collaborative analysis of Diagnostic criteria in Europe (DECODE); Emerging Risk Factor Collaboration (ERFC); Prospective Cohort Studies (PSC); American Cancer Society Cancer Preventions Study, Phase II (ACS CPS-II)

**References**

1. He FJ, et al. (2007) Increased consumption of fruit and vegetables is related to a reduced risk of coronary heart disease: meta-analysis of cohort studies. J Hum Hypertens 21(9): p. 717-28.
2. Lock K, Pomerleau J, Causer L, McKee M (2004) Low fruit and vegetable consumption. In: Ezzati M, Lopez AD, Rodgers A, Murray CJL, editors. Comparative quantification of health risks: Global and regional burden of disease attributable to selected major risk factors. Geneva: WHO. pp. 597–728.
3. Boeing H, Dietrich T, Hoffmann K, Pischon T, Ferrari P, et al. (2006) Intake of fruits and vegetables and risk of cancer of the upper aero-digestive tract: the prospective EPIC-study. Cancer Causes Control 17: 957–969.
4. Lim SS, et al. (2012) A comparative risk assessment of burden of disease and injury attributable to 67 risk factors and risk factor clusters in 21 regions, 1990-2010: a systematic analysis for the Global Burden of Disease Study 2010. The Lancet 380: 2224-2260.
5. Rehm J, Room R, Monteiro M, Gmel G, Graham K, et al. (2004) Alcohol use. In: Ezzati M, Lopez AD, Rogers A, Murray CJL, eds. Comparative quantification of health risks: Global and regional burden of disease attributable to selected major risk factors. Geneva: WHO. pp 959–1108.
6. Corrao G, Rubbiati L, Bagnardi V, Zambon A, Poikolainen K (2000) Alcohol and coronary heart disease: a meta-analysis. Addiction 95: 1505–1523.
7. Bagnardi V, Scotti L, La Vecchia C, Zatonski W (2008) Binge drinking and cardiovascular disease: a meta-analysis. J Epidemiol Community Med 62: 615–619.
8. Reynolds K, Lewis B, Nolen JD, Kinney GL, Sathya B, et al. (2003) Alcohol consumption and risk of stroke: a meta-analysis. JAMA 289: 579–588.
9. Gutjahr E, Gmel G, Rehm J (2001) Relation between average alcohol consumption and disease: an overview. Eur Addict Res 7: 117–127.
10. Ridolfo S, Stevenson C (2001) The quantification of drug-caused mortality and morbidity in Australia 1998. Canberra: Australian Institute of Health and Welfare.
11. Cho E, Smith-Warner SA, Ritz J, van den Brandt PA, Colditz GA, et al. (2004) Alcohol intake and colorectal cancer: a pooled analysis of 8 cohort studies. Ann Intern Med 140: 603–613.
12. Corrao G, Bagnardi V, Zambon A, Arico S (1999) Exploring the dose-response relationship between alcohol consumption and the risk of several alcoholrelated conditions: a meta-analysis. Addiction 94: 1551–1573.
13. Hurst PM, Harte D, Frith WJ (1994) The Grand Rapids dip revisited. Accid Anal Prev 26: 647–654.
14. Bull F, Armstrong T, Dixon T, Ham S, Neiman A, et al. (2004) Physical inactivity. In: Ezzati M, Lopez AD, Rodgers A, Murray CJL, eds. Comparative quantification of health risks: Global and regional burden of disease attributable to selected major risk factors. Geneva: WHO. pp 729–882.
15. Ezzati M, Henley SJ, Thun MJ, Lopez AD (2005) Role of smoking in global and regional cardiovascular mortality. Circulation 112: 489–497.
16. Ezzati M, Henley SJ, Lopez AD, Thun MJ (2005) Role of smoking in global and regional cancer epidemiology: current patterns and data needs. Int J Cancer 116: 963–971.
17. Thun MJ, Apicella LF, Henley SJ (2000) Smoking vs other risk factors as the cause of smoking-attributable deaths: confounding in the courtroom. JAMA 284: 706–712.
18. Lin HH, Ezzati M, Murray M (2007) Tobacco smoke, indoor air pollution and tuberculosis: a systematic review and meta-analysis. PLoS Med 4: e20.
19. O’Seaghdha CM, et al., (2009) Blood pressure is a major risk factor for renal death. An analysis of 560352 participants from the Asia-Pacific region. Hypertension 54:00-00.
20. The Renal Risk Collaboration, Foote C, Lin J, et al. The effect of Blood Pressure on Kidney Failure: a systematic review and meta-analysis in 2.7 million participants. Unpublished.
21. Renehan AG, Tyson M, Egger M, Heller RF, Zwahlen M (2008) Body-mass index and incidence of cancer: a systematic review and meta-analysis of prospective observational studies. Lancet 371: 569–578.
22. Cummings P, Wells JD, Rivara FP (2003) Estimating seat belt effectiveness using matched-pair cohort methods. Accident Analysis and Prevention 35:143-149.
23. Kelly, J.H., Jr. and J. Sabate, Nuts and coronary heart disease: an epidemiological perspective. Br J Nutr, 2006. 96 Suppl 2: p. S61-7.
