# Supplementary material for: Validation of a new predictive risk model: measuring the impact of the major modifiable risks of death for patients and populations
Source: Popul Health Metr. 2015 Oct 1;13:27. doi: 10.1186/s12963-015-0059-8 (PMC4591717; doi:10.1186/s12963-015-0059-8)

**Web Appendix C:** **Example of Risk Calculator Applied to a Particular Person: Male Age 35, Smoker, Uncontrolled Blood Pressure, Limited Physical Activity**

The table (below) shows the values that would be entered into the risk calculator for a hypothetical 35-year-old male who smokes cigarettes*, is overweight* and has elevated blood pressure.* The data display that this person would see to illustrate his current risk level (“**Your Health Risk:** **Very High**”) compared to other males in his age group is shown in the first panel and the **Interactive Risk Calculator** that can be used to see the impact of lowering or increasing different risk factors, (using slider bars) on overall avoidable risk is shown in the second panel.

| **Risk Factor** | **Hypothetical Values** |
| --- | --- |
| BMI | 34.2 |
| HbA1c | 5.2% |
| LDL | 115 mg/dL |
| Systolic Blood Pressure | 155 mm Hg |
| Alcohol Frequency (days/month) | 4 days/month |
| Alcohol Frequency (drinks/day) | 2 drinks/day |
| Binge Drinking (per month) | Never |
| Tobacco Use | current smoker |
| Vigorous Exercise | 0 days/week |
| Moderate Exercise | 3 days/week;  30 minutes/day |
| Fruit Intake (weekly) | 7 servings |
| Vegetable Intake (weekly) | 7 servings |
| Peanut Butter Intake (weekly) | 3 servings |
| Nut Intake (weekly) | 3 servings |
| Fish Intake (weekly) | 1 servings |
| Omega-3 Intake (weekly) | 2 servings |
| Seat Belt Use | always |


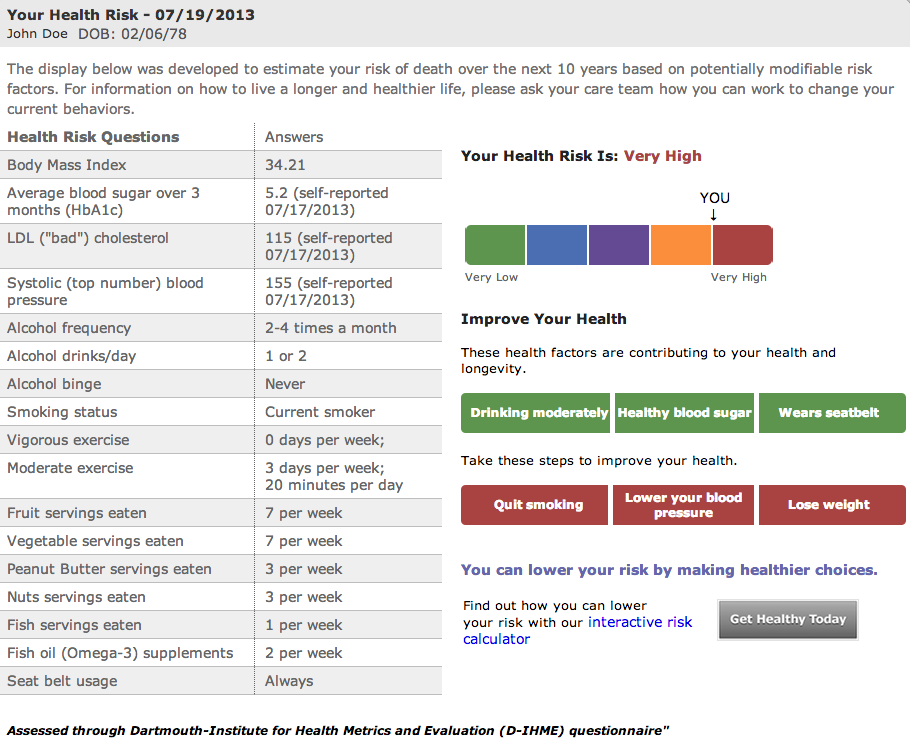

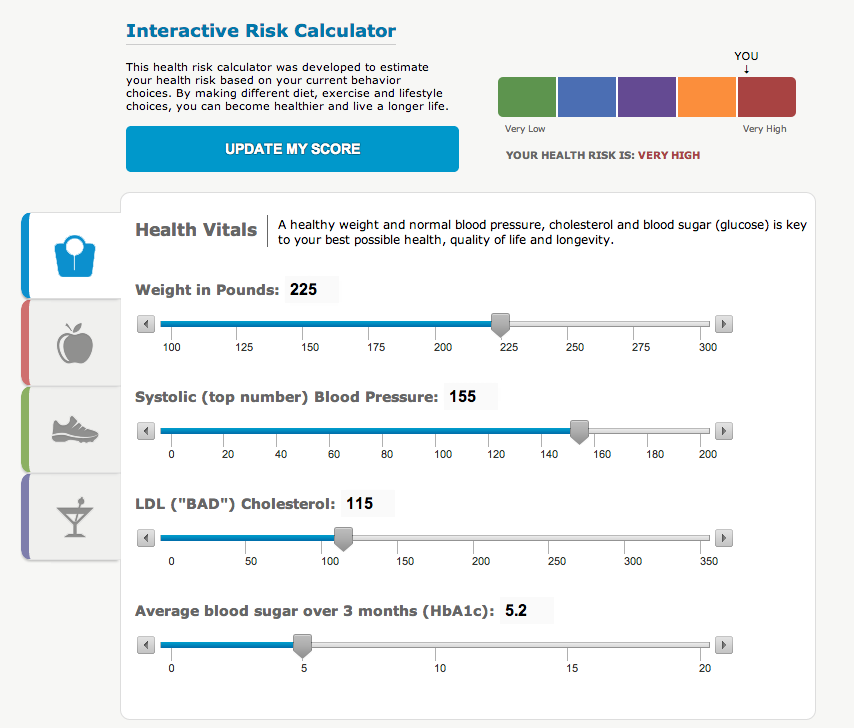

Supplement: Additional file 3: — Web Appendix C: Example of risk calculator applied to a particular person: male age 35, smoker, uncontrolled blood pressure, limited physical activity. (DOCX 246 kb) [file 12963_2015_59_MOESM3_ESM.docx]
